# Supplementary material for: Neutralization of SARS-CoV-2 spike pseudotyped virus by recombinant ACE2-Ig
Source: Nat Commun. 2020 Apr 24;11:2070. doi: 10.1038/s41467-020-16048-4 (PMC7265355; doi:10.1038/s41467-020-16048-4)
Supplement: Supplementary file 1 — Supplementary Information [file 41467_2020_16048_MOESM1_ESM.pdf]

Supplementary Information for

Neutralization of SARS-CoV-2 spike pseudotyped virus by recombinant ACE2-Ig

Lei *et al*

Supplementary Figures

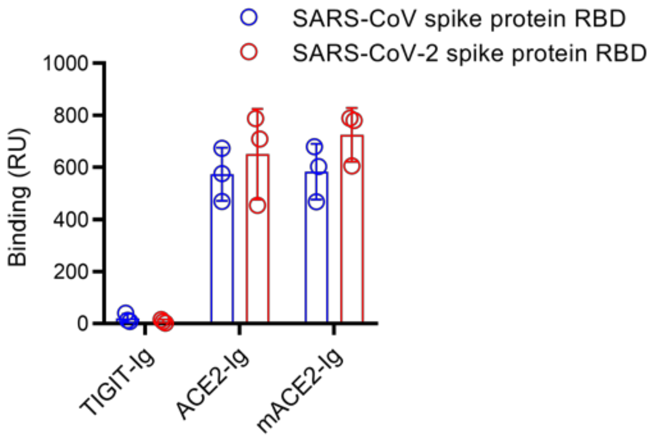

Supplementary Figure 1. Binding of Fusion proteins to CoV spike protein RBDs. Fusion proteins was tested for binding to immobilized CoV spike protein RBDs using surface plasmon resonance on a BIAcore 2000 instrument. Binding was quantified as an increase in RU at 60 s after the end of injection compared with a baseline established 20 s before injection. Data are the means ± s.d. of three independent biological replicates. Source data are provided as a Source Data file.

Figure S2

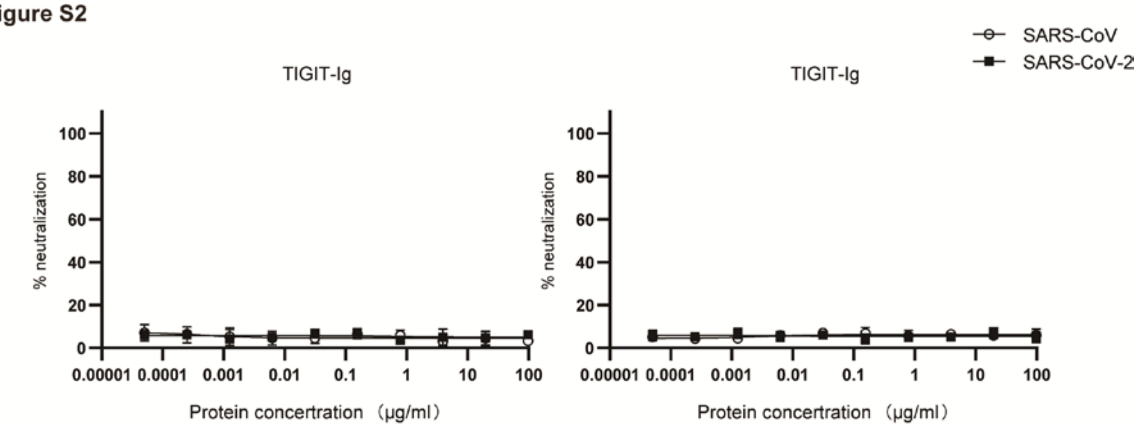

Supplementary Figure 2. No neutralization effect of TIGIT-Ig. HIVs pseudotyped with the S glycoproteins from CoVs were incubated with TIGIT-Ig for 1 h before infection. Luciferase activities in target 293T cells (left) and A549 cells (right) were measured, and the percent neutralization was calculated. Data are the means ± s.d. of four independent biological replicates. Source data are provided as a Source Data file.

Supplementary Table 1. Sequences of ACE2-Ig

Name	Amino acid sequence
ACE2-Ig	MSSSSWLLLSLVAVTAAQSTIEEQAKTFLDKFNHEAEDLFYQSSLASWN<br>YNTNITEENVQNMNNAGDKWSAFLKEQSTLAQMYPLQEIQNLTVKLQ<br>LQALQQNGSSVLSEDKSKRLNTILNTMSTIYSTGKVCNPDNPQECLLLE<br>PGLNEIMANSLDYNERLWAWESWRSEVGKQLRPLYEEYVVLKNEMAR<br>ANHYEDYGDYWRGDYEVNGVDGYDYSRGQLIEDVEHTFEEIKPLYEH<br>LHAYVRAKLMNAYPSYISPIGCLPAHLLGDMWGRFWTNLYSLTVPFGQ<br>KPNIDVTDAMVDQAWDAQRIFKEAEKFFVSVGLPNMTQGFWENSMLT<br>DPGNVQKAVCHPTAWDLGKGDFRILMCTKVTMDDFLTAHHEMGHIQY<br>DMAYAAQPFLLRNGANEGFHEAVGEIMSLSAATPKHLKSIGLLSPDFQE<br>DNETEINFLLKQALTIVGTLPFTYMLEKWRWMVFKGEIPKDQWMKKW<br>WEMKREIVGVVEPVPHDETYCDPASLFHVSNDYSFIRYYTRTLYQFQFQ<br>EALCQAAKHEGPLHKCDISNSTEAGQKLFNMLRLGKSEPWTLALENVV<br>GAKNMNVRPLLNYFEPLFTWLKDQNKNSFVGWSTDWSPYADQSIKVR<br>ISLKSALGDKAYEWNDNEMYLFRSSVAYAMRQYFLKVKNQMILFGEED<br>VRVANLKPRISFNFFVTAPKNVSDIIPRTEVEKAIRMSRSRINDAFRLNDN<br>SLEFLGIQPTLGPPNQPPVSEPKSCDKTHTCPPCPAPELLGGPSVFLFPPK<br>PKDTLMISRTPEVTCVVVDVSHEDPEVKFNWYVDGVEVHNAKTKPRE<br>EQYNSTYRVVSVLTVLHQDWLNGKEYKCKVSNKALPAPIEKTISKAKG<br>QPREPQVYTLPPSRDELTKNQVSLTCLVKGFYPSDIAVEWESNGQPENN<br>YKTTPPVLDSDGSFFLYSKLTVDKSRWQQGNVFSCSVMHEALHNHYTQ<br>KSLSLSPGK
mACE2-Ig	MSSSSWLLLSLVAVTAAQSTIEEQAKTFLDKFNHEAEDLFYQSSLASWN<br>YNTNITEENVQNMNNAGDKWSAFLKEQSTLAQMYPLQEIQNLTVKLQ<br>LQALQQNGSSVLSEDKSKRLNTILNTMSTIYSTGKVCNPDNPQECLLLE<br>PGLNEIMANSLDYNERLWAWESWRSEVGKQLRPLYEEYVVLKNEMAR<br>ANHYEDYGDYWRGDYEVNGVDGYDYSRGQLIEDVEHTFEEIKPLYEH<br>LHAYVRAKLMNAYPSYISPIGCLPAHLLGDMWGRFWTNLYSLTVPFGQ<br>KPNIDVTDAMVDQAWDAQRIFKEAEKFFVSVGLPNMTQGFWENSMLT<br>DPGNVQKAVCHPTAWDLGKGDFRILMCTKVTMDDFLTAHREMGRIQY<br>DMAYAAQPFLLRNGANEGFHEAVGEIMSLSAATPKHLKSIGLLSPDFQE<br>DNETEINFLLKQALTIVGTLPFTYMLEKWRWMVFKGEIPKDQWMKKW<br>WEMKREIVGVVEPVPHDETYCDPASLFHVSNDYSFIRYYTRTLYQFQFQ<br>EALCQAAKHEGPLHKCDISNSTEAGQKLFNMLRLGKSEPWTLALENVV<br>GAKNMNVRPLLNYFEPLFTWLKDQNKNSFVGWSTDWSPYADQSIKVR<br>ISLKSALGDKAYEWNDNEMYLFRSSVAYAMRQYFLKVKNQMILFGEED<br>VRVANLKPRISFNFFVTAPKNVSDIIPRTEVEKAIRMSRSRINDAFRLNDN<br>SLEFLGIQPTLGPPNQPPVSEPKSCDKTHTCPPCPAPELLGGPSVFLFPPK<br>PKDTLMISRTPEVTCVVVDVSHEDPEVKFNWYVDGVEVHNAKTKPRE<br>EQYNSTYRVVSVLTVLHQDWLNGKEYKCKVSNKALPAPIEKTISKAKG<br>QPREPQVYTLPPSRDELTKNQVSLTCLVKGFYPSDIAVEWESNGQPENN<br>YKTTPPVLDSDGSFFLYSKLTVDKSRWQQGNVFSCSVMHEALHNHYTQ<br>KSLSLSPGK

Supplementary Table 2. Selected analytical data and pharmacokinetic parameters of recombinant fusion proteins.

Parameter[a]	ACE-Ig	mACE2-Ig	TIGIT-Ig
HMW formation after storage(% SEC area)[b]	< 0.1	< 0.1	< 0.1
LMW formation after storage(% SEC area)[b]	< 0.1	< 0.1	< 0.1
AUC (day μg ml^{-1})	540.8	464.3	557.2
$T_{1/2}$(day)	5.2	5.3	4.7
CL (ml day^{-1} kg^{-1})	7.2	8.5	7.3
VSS(ml kg^{-1})	68.3	78.0	65.6

[a] Pharmacokinetic parameters were calculated using a noncompartmental analysis. AUC, area under the concentration versus time curve; t1/2, half-life; CL, clearance; VSS, steady-state volume of distribution.

[b] Quiescent storage for 1 wk, 40 °C, 1 mg/mL
